# Analysis of risk factors of ST-segment elevation myocardial infarction in young patients

**DOI:** 10.1186/1471-2261-14-179

**Published:** 2014-12-09

**Authors:** Wang Yunyun, Li Tong, Liu Yingwu, Liu Bojiang, Wang Yu, Hu Xiaomin, Li Xin, Peng Wenjin, JinFang Li

**Affiliations:** Cardiac Center, Third Central Hospital of Tian Jin, Tian Jin, 300170 China; Essen Medical Associates, P.C.2015 Grand concourse, Bronx, NY 10453 USA

**Keywords:** Antecedent angina pectoris, Fibrinogen, Glycated hemoglobin, Risk factors, ST-segment elevation myocardial infarction, Young patients

## Abstract

**Background:**

Acute myocardial infarction (AMI) is often present in old populations and rare in young people. Its incidence significantly increased recent years. The mechanism and disease course of AMI in young people are probably different from that in old population. The aim of this study was to analyze clinical risk factors of STEMI in young patients.

**Methods:**

Data was collected from consecutive patients ≤ 44 years of age (young; *n* = 86) and 60–74 years of age (old; *n* = 65) diagnosed with STEMI, and 79 young age-matched patients without coronary artery disease (CAD), hospitalized between January 2009 and June 2013.

**Results:**

The young STEMI group had a significantly higher proportion of males (88.37 *vs.* 53.16%; *P* < 0.01), smokers (82.56 *vs.* 49.37%; *P* < 0.01) and patients with a family history of early CAD (54.65 *vs*. 32.91%; *P* < 0.05) than age-matched controls. Young STEMI patients also had significantly higher levels of fasting blood sugar (6.39 *vs.* 5.25 mmol/L; *P* < 0.001), glycated hemoglobin (HbA1c) (6.26 *vs.* 5.45%; *P* < 0.05), total cholesterol (5.14 *vs.* 4.65 mmol/L, *P* < 0.05), and fibrinogen (Fib) (3.39 *vs.* 2.87; *P* < 0.01). Compared with the old STEMI group, young STEMI patients had significantly higher proportions of males (88.37 *vs.* 63.08%; *P* < 0.01) smokers (82.56 *vs.* 41.54%; *P* < 0.01), and those with a family history of early CAD (54.65 *vs.* 18.46%; *P* < 0.01). Young STEMI patients also lower Fib (3.39 *vs.* 3.88 g/L; *P* < 0.01), less frequent occurrence of angina pectoris before STEMI (13.95 *vs.* 29.23%; *P* < 0.05) compared with the old STEMI group. Logistic regression analysis indicated that male sex (OR = 5.891), smoking (OR = 3.500), family history of early CAD (OR = 3.194), Fib (OR = 2.414) and HbA1c (OR = 1.515) are associated with STEMI in young patients.

**Conclusion:**

In addition to previously recognized risk factors (male sex, smoking and family history of early CAD), Fib and HbA1c are associated with STEMI in individuals ≤ 44 years of age without antecedent angina pectoris.

## Background

Acute myocardial infarction (AMI) is a major cause of death worldwide. AMI is less frequent in adults younger than 45 years of age than in elderly adults [1, 2], but is of increasing clinical interest in young adults because of the potential of premature death and long-term disability [3]. The incidence of AMI in young people was as low as 2–6% [1, 2], but has recently begun to rise. The protection offered by young age is countered by increased prevalence of risk factors for coronary artery disease (CAD), such as impaired glucose tolerance and obesity, in adolescence [4]. Ignorance of CAD combined with a false sense of security likely prevents younger individuals from seeking medical advice. Early recognition and risk factor modification in this population is of key importance [5].

The mechanism and disease course of AMI in young patients are likely different from those in an older population, and knowing of theses differences may help to prevent the disease and improve the prognosis [6]. However, there are few studies investigating risk factor profiles and patterns of coronary artery involvement in ST-segment elevation myocardial infarction (STEMI) in younger individuals. This paper retrospectively analyzed risk factors and clinical features in young patients with STEMI to characterize this medical condition.

## Methods

### Ethics statement

This study was approved by ethics committee of The Third Central Hospital of Tian Jin. The study protocols comply with the ethical guidelines of the Declaration of Helsinki. All subjects provided informed consent.

### Subjects

This was a retrospective study of 86 consecutive young patients (18–44 years of age) with STEMI selected from a total of 2460 AMI patients enrolled between January 2009 and June 2013 at The Third Central hospital of Tianjin. A group of 79 young adults of the same age range who made consecutive visits to the hospital during the same period, were enrolled as controls. Coronary heart disease was excluded in this group by coronary angiography. An additional group of 65 consecutive old patients (60–74 years of age) with STEMI were included for comparison.

### Inclusion criteria

Patients included in the study were diagnosed with STEMI, defined by the typical rise and fall of cardiac markers of myocardial necrosis with at least one of the following [7]: i) symptoms of ischemia; ii) echocardiogram changes indicative of new ischemia (≥ 0.1 mV in two or more standard leads, ≥ 0.2 mV in two or more contiguous pre-cordial leads, or a new left bundle branch block); iii) 12 h after symptoms, levels of creating kinase and its isoform (CKMB) were twice the normal upper limit or a troponin level was increased to the standard of MI (according to the normal local laboratory value). All patients had complete medical records and had undergone coronary angiography.

### Exclusion criteria

Patients with the following conditions were excluded: congenital heart disease, cardiomyopathy, myocarditis, Takayasu’s arteritis or vascular dysplasia; coronary artery embolism; AMI secondary to aortic dissection, severe aorta valve stenosis, myocardial hypertrophy, and history of AMI without evidence from angiography showing narrowing of the coronary arteries.

### Data collection

Demographic and baseline data were collected by questionnaire at patient interviews and by review of medical records. Medical records were analyzed for the patients’ CAD risk factor profiles. Baseline data included gender, age, body mass index, history of smoking or drinking, family history of CAD, medical history of hypertension, type II diabetes mellitus, cranial vascular accidents, systolic and diastolic pressure, and the presence of other diseases. A TBA-120FR auto-biochemical analyzer (Toshiba, Japan) and Sysmex kx-21 hematology analyzer (Sysmex, Japan) were used to measure fasting blood sugar, glycated hemoglobin (HbA1c), creatine kinase and CKMB, troponin I (normal range 0.05–0.40 ng/mL), triglycerides (TG), total cholesterol (TC), non-high-density lipoprotein cholesterol (NHDL-C), HDL-C, and fibrinogen (Fib) in the morning after hospital admission, following an overnight fast.

### Criteria for other conditions

Hypertension was defined as a systolic pressure ≥ 140 mmHg and/or a diastolic pressure ≥ 90 mmHg [8]; a history of hypertension was noted in patients using anti-hypertension medications. Type II diabetes was noted in patients who met the 1999 WHO diagnosis criteria [9]. Unstable angina pectoris (AP) before AMI included initial AP on effort, exacerbation of effort AP, spontaneous AP, mixed AP, variant angina, and angina that occurred within one month of AMI, each episode lasting 2–20 min [10]. Smoking was defined as smoking for six months or longer, and a smoking index was calculated as the number of daily cigarettes × years of smoking [11]. A family history of early CAD was recorded in patients where it was first diagnosed in the father at ≤ 55 or the mother at ≤ 65 years of age.

### Coronary angiography

Due to delays for hospital admittance in some patients, coronary angiography was performed within one month after STEMI via the radial artery using a multifunction catheter or via the femoral artery using the Judkins method. The results were interpreted by two experienced physicians.

### Statistical analysis

SPSS 17.0 software (SPSS Inc., Chicago, IL, USA) was used for all data analysis. Normally distributed numeric data are presented as means ± standard deviation and intergroup comparisons were conducted with a Student’s *t*-test. Non-normally distributed numeric data are presented as medians and quartiles (M [Q1, Q3]) and intergroup comparisons were conducted with the Mann–Whitney *U* test. Categorical data were tested with the *χ*^2^ test. A logistic regression model was used to identify risk factors of STEMI in young patients. All tests were two sided and *P* < 0.05 was regarded as significant.

## Results

### Comparison between young STEMI and control patients

Baseline clinical and laboratory test data in the young STEMI and young control groups are summarized in Table 1. The proportions of smokers and patients with a family history of early CAD, and the smoking index values in the STEMI group were significantly higher than those in the control group (*P*s < 0.05). STEMI patients had significantly higher fasting blood sugar, HbA1c, TC, NHDL-C and Fib levels than controls (*P*s < 0.05). There were no differences between the two groups in age, blood pressure, TG, HDL-C, hypertension, type II diabetes, cerebrovascular diseases, or drinking.

**Table 1:** **Clinical data from the young groups**

Variable	STEMI	Control	***P***
	(***n*** = 86)	(***n*** = 79)	
Age, y	40.00 (23, 44)	39.94 (27, 44)	0.317
Male, *n* (%)	76 (88.37)	42 (53.16)	<0.001
BMI (kg/m^2^)	27.96 ± 2.80	25.16 ± 3.15	0.127
Systolic pressure (mmHg)	133.59 (90, 200)	134.82 (100, 190)	0.629
Diastolic pressure (mmHg)	81.03 (50, 124)	83.37 (60, 140)	0.957
Pulse pressure (mmHg)	52.67 (25, 90)	51.71 (30, 90)	0.608
Fasting blood sugar (mmol/L)	6.39 (3.76, 18.00)	5.25 (3.25, 12.35)	<0.001
HbA1C (%)	6.26 ± 1.69	5.45 ± 1.12	0.018
CK (U/L)	1950.28 ± 1639.52	81.15 ± 44.60	<0.001
CKMB (U/L)	163.99 ± 127.38	14.77 ± 13.35	<0.001
TNI (ng/mL)	3.08 (0.11, 30)	0.07 (0.05, 0.3)	<0.001
TG (mmol/L)	2.37 ± 2.48	2.25 ± 1.26	0.201
TC (mmol/L)	5.14 ± 1.39	4.65 ± 0.87	0.002
NHDL-C (mmol/L)	4.06 ± 1.35	3.53 ± 0.87	0.002
HDL-C (mmol/L)	1.07 ± 0.27	1.12 ± 0.26	0.856
Fib (g/L)	3.39 (1.90, 7.52)	2.87 (1.78, 5.30)	<0.001
Hypertension, *n* (%)	41 (47.67)	35 (44.30)	0.755
Type II diabetes, *n* (%)	18 (20.93)	17 (21.52)	1.000
Cerebrovascular diseases, *n* (%)	6 (6.98)	2 (2.53)	0.281
Alcohol drinking, *n* (%)	25 (29.07)	19 (24.05)	0.486
Smoking, *n* (%)	71 (82.56)	39 (49.37)	<0.001
Smoking index	329.53 (0, 1200)	179.49 (0, 900)	<0.001
Family history of early CAD, *n* (%)	47 (54.65)	26 (32.91)	0.007

*Abbreviations*: *BMI* body mass index, *CAD* coronary artery disease, *CK* creatine kinase, *CKMB* isoform of CK, *Fib* fibrinogen, *HbA1c* glycated hemoglobin, *HDL-C* high-density lipoprotein cholesterol, *MI* myocardial infarction, *NHDL-C* non-HDL-C, *TC* total cholesterol, *TG* triglyceride, *TNI* troponin I, *STEMI* ST-segment elevation myocardial infarction.

Note: Data are expressed as mean ± standard deviation or medians (Q1, Q3).

### Comparison between young and old STEMI patients

Baseline clinical and laboratory test data in the young and old STEMI groups are summarized in Table 2. The gender, diastolic pressure, pulse pressure, smoking, smoking index values, and family history of early CAD were significantly different between the two groups (*P*s < 0.05). Patients in the young group were less likely to have cerebrovascular diseases or experience AP before STEMI than the older patients. TG, TC, and NHDL-C levels were higher, and serum Fib was lower, in young compared with old STEMI patients (*P*s < 0.05). Smoking and imbalance of lipid metabolism were more common in young STEMI patients than in old patients (*P*s < 0.05).

**Table 2:** **Comparison of young and old STEMI groups**

Variable	Young	Old	***P***
	(***n*** = 86)	(***n*** = 65)	
Age, y	40.00 (23, 44)	69.15 (63,74)	< 0.001
Male, *n* (%)	76 (88.37)	41 (63.08)	0.001
BMI, kg/m^2^	27.96 ± 2.80	26.18 ± 2.30	0.346
Systolic pressure, mmHg	133.59 (90, 200)	131.09 (96,180)	0.885
Diastolic pressure, mmHg	81.03 (50, 124)	73.20 (46, 120)	0.005
Pulse pressure, mmHg	52.67 (25, 90)	57.89 (30, 100)	0.030
Fasting blood sugar (mmol/L)	6.39 (3.76, 18.00)	6.02 (3.61, 12.00)	0.682
HbA1C (%)	6.26 ± 1.69	6.29 ± 1.47	0.458
CK (U/L)	1950.28 ± 1639.52	1691.35 ± 1411.02	0.533
CKMB (U/L)	163.99 ± 127.38	189.14 ± 173.26	0.066
TNI (ng/mL)	3.08 (0.11, 30)	3.40 (0.17, 30)	0.664
TG (mmol/L)	2.37 ± 2.48	1.29 ± 0.48	0.001
TC (mmol/L)	5.14 ± 1.39	4.74 ± 0.85	0.005
NHDL-C (mmol/L)	4.06 ± 1.35	3.60 ± 0.76	0.003
HDL-C (mmol/L)	1.07 ± 0.27	1.14 ± 0.24	0.760
Fib (g/L)	3.39 (1.90, 7.52)	3.88 (2.06, 10.94)	< 0.001
Hypertension, *n* (%)	41 (47.67)	35 (53.85)	0.512
Type II diabetes, *n* (%)	18 (20.93)	21 (32.30)	0.135
Cerebrovascular diseases, *n* (%)	6 (6.98)	18 (27.69)	< 0.001
Alcohol drinking, *n* (%)	25 (29.07)	7 (10.77)	0.008
Smoking, *n* (%)	71 (82.56%)	27 (41.54)	< 0.001
Smoking index	329.53 (0, 1200)	263.54 (0, 2400)	< 0.001
Family history of early CAD, *n* (%)	47 (54.65)	12 (18.46)	< 0.001
Angina pectoris before MI, *n* (%)	12 (13.95)	19 (29.23)	0.026

*Abbreviations*: *BMI* body mass index, *CAD* coronary artery disease, *CK* creatine kinase, *CKMB* isoform of CK, *Fib* fibrinogen, *HbA1c* glycated hemoglobin, *HDL-C* high-density lipoprotein cholesterol, *MI* myocardial infarction, *NHDL-C* non-HDL-C, *STEMI* ST-segment elevation myocardial infarction, *TC* total cholesterol, *TG* triglyceride, *TNI* troponin I.

Note: Data are expressed as mean ± standard deviation or medians (Q1, Q3).

### Risk factors of STEMI

Using STEMI as a dependent variable, independent risk factors among young patients were determined as those with a *P* < 0.25 in Student’s *t*-tests, including gender, smoking history, smoking index, family history of early CAD, and levels of blood sugar, HbA1c, TC, NHDL-C, and Fib, and assessed by a logistic regression analysis. The analysis revealed that gender, smoking, family history of CAD, Fib, and HbA1c levels were independent risk factors for STEMI in young individuals (*P*s < 0.05) (Table 3).

**Table 3:** **Logistic regression analysis of STEMI risk factors in young patients**

Factors	B value	SE	Wald	OR	95% CI	***P***
Male	1.7731	0.508	12.177	5.891	2.176–15.950	<0.001
Smoking history	1.253	0.473	7.016	3.500	1.385–8.842	0.008
Family history of early CAD	1.161	0.409	8.059	3.194	1.433–7.122	0.005
Fib	0.881	0.288	9.366	2.414	1.373–4.245	0.002
HbA1c	0.415	0.165	6.370	1.515	1.097–2.091	0.012

*Abbreviations*: *CAD* coronary artery disease, *CI* confidence interval, *Fib* fibrinogen, *HbA1c* glycated hemoglobin, *OR* odds ratio, *SE* standard error, *STEMI* ST-segment elevation myocardial infarction.

## Discussion

The lifestyles of young people, characterized by high work stress, fast pace, overwork, smoking, drinking alcohol, and overeating, likely cause disturbances in the internal environment, such as coronary atherosclerosis, that increase the incidence of AMI [12]. Atherosclerosis, which is affected by many factors, may cause coronary spasms or broken plaque in coronary arteries resulting in acute blockage [13]. In this study, we found that STEMI tended to occur suddenly in young patients and in those without a history of AP.

Conventional risk factors for STEMI include male sex, smoking, and a family history of early CAD [14]. In accordance with this and another study [15], we found that male sex was an important risk factor for STEMI in young patients. Androgen was shown to negatively correlate with the incidence of STEMI, and physiologic levels can prevent atherosclerosis [16]. Androgen levels, which peak at age 20–24 and then decline gradually, are significantly reduced in atherosclerosis patients, and low androgen levels can induce heart diseases and predict AMI [17]. The unhealthy habits such as smoking, drinking, and eating high-fat or high-purine diets may also increase the risk of AMI in young men compared with older male and young female populations. This may explain why the group of young STEMI patients included a significantly higher proportion of males than the old group.

A recent study published by Cases and Rate [18] found that among 6892 STEMI who received percutaneous coronary intervention, 46.4% were smokers, compared with 20.5% in the general population. Studies in China and other countries demonstrated that young AMI patients have smoking rates as high as 70–90% [5, 19]. As our data show, young STEMI patients are more likely than older patients to be smokers. The risk of AMI decreases after smokers quit, and the benefit of quitting is correlated with amount smoked [11]. Moreover, smoking cessation can help prevent cardiovascular events, especially in young people [18].

The results of our study show that 58.33% of young patients have a family history of early CAD, which is higher than the 30–40% of young AMI patients reported by Colkesen *et al.* [20]. Patients with a family history of CAD have more severe disease progression than those without a history [21], more lipid metabolism disorders, and more likely to have insulin resistance and be obese, possibly resulting from hereditary factors [22].

An important finding from our study is that levels of HbA1c and Fib were independent risk factors for STEMI in young patients. Fib is associated with vascular endothelial injury, and enhances the coagulation of platelets and increase in blood viscosity to induce thrombosis. Fib level is significantly associated with coronary artery calcification and sclerosis [23], and levels are significantly higher in patients who die from coronary diseases than in those who survive [24]. A study by Tatli *et al.* [25] showed that Fib can predict the extent of coronary artery narrowing in young AMI patients. In this study, the Fib levels were significantly higher in young STEMI patients compared with controls, and logistic regression analysis indicated it was an independent risk factor of STEMI, further confirming the role of coagulation disorders in STEMI in the young population. Old STEMI patients also had higher Fib levels, suggesting that its effect in this population is even more important.

Although diabetes is an important risk factor for coronary disease, its incidence was not significantly higher in the young versus the old STEMI patients. However, many studies report that non-diabetic AMI patients have increased blood sugar, compromised glucose tolerance, and insulin resistance [26, 27]. There is a significant correlation between HbA1c level, an indicator of long-term glycemic control, and the development and prognosis of coronary diseases; this correlation is less frequently reported in young patients [28]. In our study, STEMI patients were younger than the peak age of diabetes incidence, though baseline fasting blood sugar and HbA1c were significantly higher, suggesting that a higher proportion of patients had undetected diabetics or pre-diabetes. As pre-diabetic conditions can influence the course of STEMI, medical intervention in young people may help prevent STEMI. The findings of this study show that whereas fasting blood sugar level is a dependent risk factor, HbA1c is an independent risk factor, indicating that HbA1c is more strongly correlated with STEMI in young people.

Unstable AP before AMI is a clinical manifestation of ischemic preconditioning; repeated AP prepares the myocardium, reducing myocardial injury, cardiac dysfunction and severe cardiac arrhythmia following AMI [29]. Klein *et al.* [30] found that young patients seldom experience AP before MI, but that AP quickly progresses to AMI. Consistent with their findings, only 12% of the young STEMI patients in our study experienced AP before STEMI, a significantly smaller percentage than in old STEMI patients. STEMI in young patients generally has no ischemic preconditioning, and occurs and progresses faster than it does in older patients. However, troponin I values were similar between the young and old STEMI groups, and there were no differences in the degree of myocardial necrosis.

### Study limitations

This study is subject to the usual limitations associated with a retrospective design. Because of the low incidence of STEMI in young people, the sample size of this study was small. Despite adjusting for multiple risk factors, it is possible that there may have been residual confounding conditions and medications. In addition, no data were collected about coronary artery disease extension. Therefore, the influence of some confounders and biases cannot be completely excluded. Multicenter studies with large sample sizes will further elucidate the mechanism of STEMI in the young population. Nevertheless, this study provides a clinical reference for healthcare professionals to more fully understand risk factors in young STEMI patients and to develop early preventative interventions.

## Conclusion

In conclusion, STEMI in young people has some clinical features that are different from those in older patients. In addition to the conventional risk factors, Fib and HbA1c are associated with the initiation of STEMI, and can provide inexpensive and powerful prognostic factors for STEMI in young patients. STEMI in young patients is often accompanied by disorders of glucose metabolism and abnormal coagulation, though AP before STEMI is rare.

## References

[1] Fournier J, Sanchez A, Quero J, Fernandez-Cortacero J, González-Barrero A (1996). Myocardial infarction in men aged 40 years or less: a prospective clinical-angiographic study. Clin Cardiol.

[2] Garoufalis S, Kouvaras G, Vitsias G, Perdikouris K, Markatou P, Hatzisavas J, Kassinos N, Karidis K, Foussas S (1998). Comparison of angiographic findings, risk factors, and long term follow-up between young and old patients with a history of myocardial infarction. Int J Cardiol.

[3] Weinberger I, Rotenberg Z, Fuchs J, Sagy A, Friedmann J, Agmon J (1987). Myocardial infarction in young adults under 30 years: risk factors and clinical course. Clin Cardiol.

[4] Sinha R, Fisch G, Teague B, Tamborlane WV, Banyas B, Allen K, Savoye M, Rieqer V, Taksali S, Barbetta G, Sherwin RS, Caprio S (2002). Prevalence of impaired glucose tolerance among children and adolescents with marked obesity. N Engl J Med.

[5] Jamil G, Jamil M, Alkhazraji H, Haque A, Chedid F, Balasubramanian M, Khairallah B, Qureshi A (2013). Risk factor assessment of young patients with ST-segment elevation myocardial infarction. Am J Cardiovasc Dis.

[6] Egred M, Viswanathan G, Davis G (2005). Myocardial infarction in young adults. Postgrad Med.

[7] Chinese Society of Cardiology of Chinese Medical Association, Editorial committee of Chinese Journal of Cardiology, Editorial committee of Chinese Circulation Journal (2001). Acute myocardial infarction diagnosis and treatment guidelines. Chin J Cardiol (Chin).

[8] Chinese Hypertension Prevention and Treatment Guide Revision Committee (2004). Chinese hypertension prevention and treatment guide 2004. Chin J Cardiol.

[9] Ye RG, Lu ZY, Xie Y, Wang C (2006). Internal Medicine.

[10] Yang YJ, Hua W, Gao RL (2006). Fuwai Cardiovascular Medicine Manual.

[11] Zhao J, Hu D-y, Ding R-j, Li XB, Zhang P, Wang L, Yu XJ, Guo JH, Wang XQ, Li L, Zhang FF, Huang ZW (2010). Coronary characteristics of young smokers with coronary heart disease and the effects of tobacco control on smoking cessation [J]. Zhonghua Yi Xue Za Zhi.

[12] Wang XY (2012). Analysis of 56 young patients with acute myocardial infarction. J Med Theory Pract.

[13] Ma CT, Jiang YX, Du WJ, Liu ZF, Wang J (2012). The correlation analysis of serum BNP, hypersentive C-creative protein and left ventricular ejaction fraction of acute myocardial infarction patients. Chin Crit Care.

[14] Schoenenberqer AW, Radovanovic D, Stauffer JC, Windecker S, Urban P, Niedermaier G, Keller PF, Gutzwiller F, Erne P (2011). Acute coronary syndromes in young patients: presentation, treatment and outcome. Int J Cardiol.

[15] Egiziano G, Akhtari S, Pilote L, Daskalopoulou S (2013). Sex differences in young patients with acute myocardial infarction. Diabet Med.

[16] Provotorov V (2007). Age-related androgen deficiency in men with ischemic heart disease. Adv Gerontol.

[17] Zhang X, Li X, Cao T, Ye L (2011). Correlation of endogenous androgen and androgen receptor level with coronary artery diseases in elderly males]. Zhonghua Yi Xue Za Zhi.

[18] Cases N, Rate ES (2013). The ongoing importance of smoking as a powerful risk factor for ST-segment elevation myocardial infarction in young patients. JAMA.

[19] Barbash G, White H, Modan M, Diaz R, Hampton J, Heikkila J, Kristinsson A, Moulopoulos S, Paolasso E, Werf TV (1995). Acute myocardial infarction in the young-the role of smoking. Eur Heart.

[20] Colkesen AY, Acil T, Demircan S, Sezgin AT, Muderrisoglu H (2008). Coronary lesion type, location, and characteristics of acute ST elevation myocardial infarction in young adults under 35 years of age. Coron Artery Dis.

[21] Gaeta G, De Michele M, Cuomo S, Guarini P, Foglia MC, Bond MG, Trevisan M (2000). Arterial abnormalities in the offspring of patients with premature myocardial infarction. N Engl J Med.

[22] Berenson GS, Srinivasan SR, Bao W, Newman WP, Tracy RE, Wattigney WA (1998). Association between multiple cardiovascular risk factors and atherosclerosis in children and young adults. N Engl J Med.

[23] Bielak LF, Klee GG, Sheedy PF, Turner ST, Schwartz RS, Peyser PA (2000). Association of fibrinogen with quantity of coronary artery calcification measured by electron beam computed tomography. Arterioscler Thromb Vasc Biol.

[24] Meade T, Chakrabarti R, Haines A, North W, Stirling Y, Thompson S, Brozović M (1980). Haemostatic function and cardiovascular death: early results of a prospective study. Lancet.

[25] Tatli E, Ozcelik F, Aktoz M (2009). Plasma fibrinogen level may predict critical coronary artery stenosis in young adults with myocardial infarction. Cardiol J.

[26] Tandjung K, van Houwelingen KG, Jansen H, Basalus MW, Sen H, Löwik MM, Stoel MG, Louwerenburg JHW, de Man FH, Linssen G (2012). Comparison of frequency of periprocedural myocardial infarction in patients with and without diabetes mellitus to those with previously unknown but elevated glycated hemoglobin levels (from the TWENTE trial). Am J Cardiol.

[27] Lazzeri C, Valente S, Chiostri M, Picariello C, Attanà P, Gensini GF (2012). Glycated hemoglobin in ST-elevation myocardial infarction without previously known diabetes: Its short and long term prognostic role. Diabetes Res Clin Pract.

[28] Timmer JR, Hoekstra M, Nijsten MW, van der Horst IC, Ottervanger JP, Slingerland RJ, Dambrink J-HE, Bilo HJ, Zijlstra F, van’t Hof AW (2011). Prognostic value of admission glycosylated hemoglobin and glucose in nondiabetic patients with ST-segment–elevation myocardial infarction treated with percutaneous coronary intervention. Circulation.

[29] Przyklenk K, Whittaker P (2000). Brief antecedent ischemia enhances recombinant tissue plasminogen activator–induced coronary thrombolysis by adenosine-mediated mechanism. Circulation.

[30] Klein LW, Agarwal JB, Herlich MB, Leary TM, Helfant RH (1987). Prognosis of symptomatic coronary artery disease in young adults aged 40 years or less. Am J Cardiol.

[CR31] The pre-publication history for this paper can be accessed here:http://www.biomedcentral.com/1471-2261/14/179/prepub

